# Convenient and Efficient Fabrication of Colloidal Crystals Based on Solidification-Induced Colloidal Assembly

**DOI:** 10.3390/nano9040575

**Published:** 2019-04-09

**Authors:** Ting Shao, Laixi Sun, Chun Yang, Xin Ye, Shufan Chen, Xuan Luo

**Affiliations:** Research Center of Laser Fusion, China Academy of Engineering Physics, Mianyang 621900, China; shaot05@163.com (T.S.); sunlaixi@126.com (L.S.); yangc001166@163.com (C.Y.); xyecaep@mail.ustc.edu.cn (X.Y.); chenlinxiao00@163.com (S.C.)

**Keywords:** colloidal crystal, nanoparticles, solidification-induced, colloidal crystallization

## Abstract

The simple yet efficient and versatile fabrication of colloidal crystals was investigated based on the solidification-induced colloidal crystallization process with particle/water suspension as precursor. The resulting colloidal crystals were constituted by crystal grains with sizes ranging from several tens of micrometers to a few millimeters. Each of the grains had a close-hexagonal array of colloids, which endowed the bulk colloidal crystal powders with some specific optical properties. The freezing of water was shown as the major driving force to form colloidal crystal grains, which supersaturated the solution with nanoparticles and thus induced the formation and growth of colloidal crystal seeds. This process is intrinsically different from those conventional methods based on shearing force, surface tension, columbic interaction or magnetic interaction, revealing a new strategy to fabricate colloidal crystals in a convenient and efficient way.

## 1. Introduction

Photonic crystals (PCs) are periodic structures with a refractive index variation on the scale of the light wavelength. Since the pioneering work of Yablonovitch and John [1,2,3], much relevant research has been carried out because of the unique properties, such as photonic bandgap, which can prevent light with some certain wavelengths from propagating through the crystals [4]. Among the wide variety of PC forms, the ordered assembly of colloidal nanoparticles, which is known as colloidal crystals, is one of the most promising PC materials [5,6,7]. They have gained increasing attention of researchers during the past decades not only because of the curiosity about the fundamental science of their formation [8,9] but also due to their potential applications in bioassays [10,11], sensors and displays [12,13], security identification [14], anticounterfeiting [15], light trapping in solar cell [16,17] and so on. Besides, colloidal crystals play an important role in the fabrication of inverse opal materials, where they are used as templates [18,19,20].

An essential step towards the application of colloidal crystals is the convenient and efficient fabrication. Over the past years, various methods have been investigated for the preparation of colloidal crystals, which are mostly based on the self-assembly of colloidal nanoparticles. Among the most commonly-used methods, gravitational sedimentation is the earliest, which utilizes gravitational force to sink the particles to the substrate at a very slow rate, forming colloidal crystals [21,22,23,24]. In some cases, centrifugation is introduced to increase the sedimentation rate of the particles [25]. This method is easy to be practiced and is still popular nowadays. However, it is time-consuming because several days are usually needed to accomplish this process. In addition, crystal formation can only occur at specific volume fractions of colloids. Another extensively-used method is vertical deposition pioneered by Colvin et al. [26,27,28,29]. In this method, a meniscus formed between the vertical or inclined [30] substrate and the colloidal suspension is slowly swept across the substrate by solvent evaporation. Strong capillary force at the meniscus induces the ordered assembly of spheres into thin planar opals depositing on the substrate. This method is also quite time-consuming where several days are often necessary especially when multilayered colloidal crystals are prepared. Meniscus and capillary force are also utilized in droplet evaporation method to induce colloidal assembly for fabricating colloidal crystal spheres [31,32,33], where the droplet acts as a confinement of the colloids. Langmuir–Blodgett is another broadly-used method where the colloids float and form a monolayer film on a liquid surface. The floating particles assemble into a close-packed arrangement to reduce the available surface area and thus the total surface tension as the film is compressed, which is then “scooped” to a substrate [16,17,34]. Electrostatics can be used to assist the spontaneous assembling process for increasing the fabrication speed [35]. Another powerful technique is the melt-shear organization, which involves the compression of hard core–soft shell particles between the plates with heating [36,37,38]. This method permits the preparation of large-area freestanding opal films in one single step. However, it is restricted to hard core–polymer shell particles. Besides these traditional methods, external electric fields or magnetic fields are introduced in some cases to induce the self-assembly of charged colloidal particles or magnetic particles [39,40]. Each of these methods has its own advantages and disadvantages. In general, they have some common drawbacks, such as careful and precise control over the process, time-consuming or low throughput. Therefore, the investigation of new method to fabricate colloidal crystals in a simple, high-throughput and flexible way is still desired.

In this work, we developed a convenient and efficient method for preparing colloidal crystals through monodispersed-nanoparticle crystallization induced by the freezing of surrounding water. The products consisted of colloidal crystal grains with sizes ranging from several tens of micrometers to a few millimeters, and each of the grains had a close-hexagonal array of spheres. The fabrication process is very convenient and high-throughput with no need of careful control in manipulation. Besides, the strategy proposed here is intrinsically different from those conventional methods based on shearing force, surface tension, columbic interaction or magnetic interaction.

## 2. Materials and Methods

### 2.1. Preparation of Particle/Water Precursors

The monodispersed silica nanoparticles with diameters about 240 nm were synthesized using the Stöber method. The product was first washed with ethanol to remove impurities and unreacted tetraethoxysilane (TEOS) and then washed with ultrapure water (18.2 MΩ·cm^−1^) to remove ethanol by repeated centrifugation and ultrasonic dispersion cycles. SiO_2_ colloidal suspensions with different volume fractions of particles (i.e., 100% c0, 80% c0, 60% c0, 40% c0, 20% c0, where c0 is the volume fraction of SiO_2_ particles in the originally prepared suspension) were prepared by quantitatively dispersing the silica particles in ultrapure water through sonication. The monodispersed carboxyl polystyrene (COOH–PS) latex particles with certain amounts of carboxyl groups covering the particle surface were purchased from Duke Scientific Corp., Palo Alto, CA, USA. They were diluted with ultrapure water to certain volume fractions.

### 2.2. Fabrication and Characterization of Colloidal Crystals

**Figure 1:**
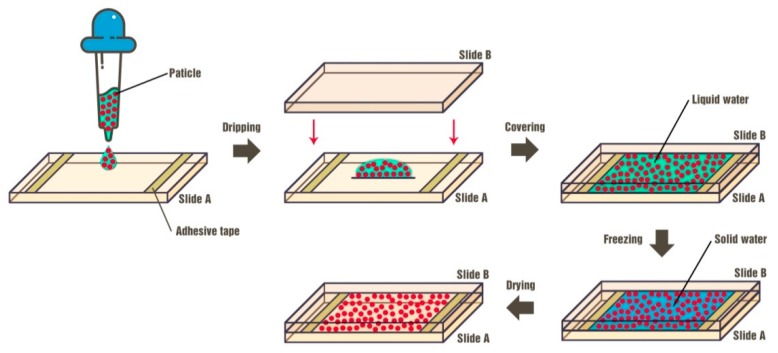
Schematics of the fabrication procedures of colloidal crystal films used for spectral measurement.

The latex suspensions (i.e., particle/water precursors) were frozen using liquid nitrogen, where the glass petri dish containing the latex suspension was placed near the surface of the liquid nitrogen in a closed environment. The suspensions solidified in a couple of minutes (with a cooling rate higher than −10 °C·min^−1^) and then were instantly transferred to the freeze-drying chamber (Epsilon 2-4 LSC, Christ). The solid water in the frozen suspensions was removed by freeze-drying, producing porous blocks comprising of colloidal-crystal grains. The products were characterized morphologically by an optical microscope (SteReo Discovery. V20, Zeiss, Jena, Germany) equipped with white light as illumination. The microstructures of the colloidal-crystal grains were examined using a scanning electron microscope (SEM, JSM-7401, Zeiss, Jena, Germany), where a thin layer of Au was sputtered on the samples prior to imaging. The reflectance spectra of the products were collected with various incident angles using a variable-angle spectroscope (Lambda 900 spectrometer, PerkinElmer, Waltham, MA, USA). Since the products were constituted by many small pieces of colloidal crystals, the appearance is pale with deterioration in visibility caused by strong scattering, which makes the characteristic reflectance spectra of the crystal hard to be detected. To facilitate the measurements, thin films composed of colloidal crystal grains were prepared according to the procedures shown in Figure 1. The transparent glass slides (76 mm × 26 mm × 1 mm) were pretreated with oxygen plasma (PDC-MG, Mingheng, China) to make it more hydrophilic for uniform spreading of the aqueous suspensions. A 100 μL colloidal suspension was dropped on one glass slide (Slide A). Then, another glass slide (Slide B) was carefully placed on top of Slide A, with the aqueous suspension filling the space between the two slides. Adhesive tapes were used to control the thickness of the liquid film, as illustrated in Figure 1. The last two steps were freezing and drying, respectively, which were the same as the preparation of colloidal crystal blocks.

## 3. Results and Discussion

**Figure 2:**
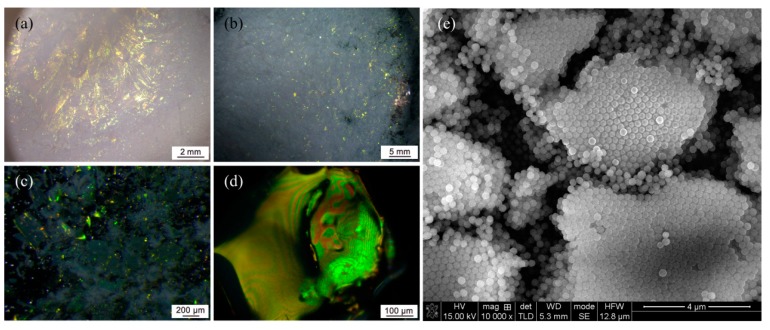
Photographs of the product: (**a**–**d**) microscopic photographs with different magnifications under white illumination; and (**e**) scanning electron microscope (SEM) photographs.

Figure 2a–d presents microscopic photographs of the product fabricated with SiO_2_ particles with diameters about 240 nm. Vivid colors were observed from the surface of the porous block with a low magnification of the optical microscopy (Figure 2a). Crushing the block into powder led to many small crystal-like grains with sizes ranging from several tens of microns to a few millimeters that reflected green light, as reported in Figure 2b,c. The magnified microscopic photograph of the crystal-like grains is shown in Figure 2d, which exhibited a brilliant green color. Since the products contained only chemically-colorless SiO_2_, the colors shown in Figure 2 should be structural colors that arose from light diffraction of the ordered arrays of the SiO_2_ colloidal particles. The array fashion of the colloidal particles was directly investigated by SEM, as shown in Figure 2e. Several fragments had random hexagonal closed pack structures of spherical particles. The cracks between these fragments probably resulted from the fracture of the colloidal crystal pieces during the preparation of the SEM samples.

**Figure 3:**
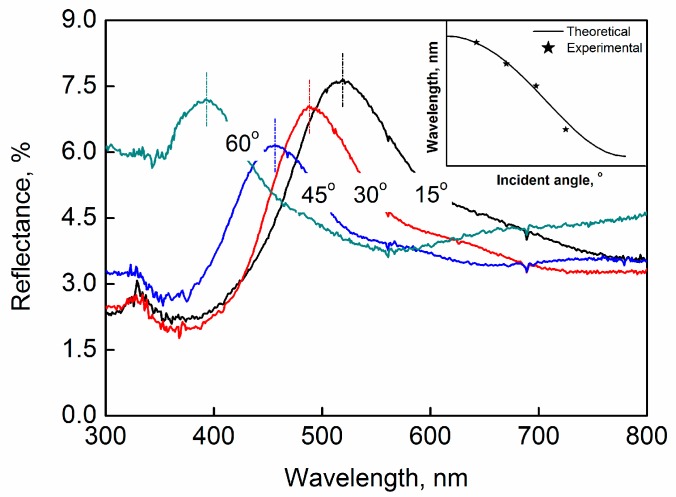
Reflection spectra of the colloidal crystal films fabricated with SiO_2_ nanoparticles (with diameters about 240 nm) with different incident angles.

To characterize the optical properties of the colloidal crystals, the reflectance spectra of the films fabricated according to the procedures shown in Figure 1 were measured. The reflectance of the SiO_2_ colloidal crystal films as a function of wavelength with different incident angles is shown in Figure 3. As shown in the figure, the reflectance had peak values at the wavelengths of 519, 488, 456 and 393 nm, with incident angles of 15°, 30°, 45° and 60°, respectively. As the incident angle increased, the peak position *λ* had a blue shift. The inserted graph in Figure 3 shows the plotting of the peak wavelengths *λ*_peak_ as a function of incident angle. Based on Bragg’s equation, *λ*_peak_ for hexagonally ordered-arranged particles under study can be estimated as:

(1) $$\lambda =d{\left(\frac{\pi }{3\sqrt{2}\varphi }\right)}^{1/3}{\left(\frac{8}{3}\right)}^{1/2}{\left(n_{p}{}^{2}\varphi +n_{m}{}^{2}(1-\varphi )-\sin^{2}\theta \right)}^{1/2}$$

where *d* is the particle diameter; *φ* is the volume fraction of particles; *n*_m_ and *n*_p_ are the refractive indices of medium and particle, respectively; and *θ* is the incident angle. Assuming the particles comprising the colloidal crystal grains were closely packed, an analytical curve describing the wavelength *λ*_peak_ as a function of incident angle *θ* was obtained according to Equation (1). Clearly, the experimental dots were near the theoretical line, implying that the reflection peaks of the film stemmed from the diffraction of the hexagonally closely packed particles. It is shown that the optical properties of the film arose from the colloidal crystal grains, which acted as building blocks.

**Figure 4:**
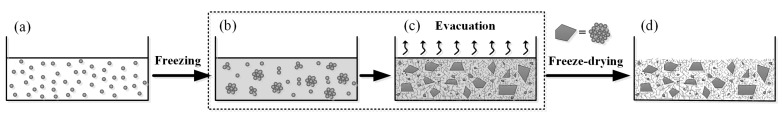
Schematics of the formation mechanism of the colloidal crystals in our method. (**a**) Well dispersed colloidal suspension; (**b**) precipitation of the nanoparticles; (**c**) colloidal crystal grains dispersed in solid water; (**d**) porous block comprising of colloidal crystal grains after the removal of solid water.

The results presented above indicate that the porous blocks or films fabricated with our method were constituted by colloidal crystal grains with sizes ranging from several tens of microns to a few millimeters. The particles comprising the grains were hexagonally closely arranged. Based on these results, the formation mechanism is illustrated in Figure 4. The particles were well dispersed in the liquid water at the beginning. As the liquid dispersion was cooled down to below 0 °C, the water began to solidify into ice. As more and more water solidified, the volume fraction of the particles suspended in the remained liquid water became higher and higher, until the liquid dispersion reached its saturated state. Some particles precipitated out of the water due to supersaturation, forming a mixture of tiny colloidal crystal seeds, partially solidified water and highly concentrated particles (Figure 4b). As the water was further frozen, the volume of liquid water decreased further and more particles precipitated out to deposit onto preformed colloidal crystal seeds or form new seeds. The preformed seeds grew larger as new particles deposit on them. When the water was totally solidified, all particles were forced to precipitate out, generating many colloidal crystal grains with sizes ranging from several tens of micrometers to a few millimeters surrounded by the solid water (Figure 4c). During this process, there was a balance between the entropically favored close-packed arrangements and the electrostatically driven non-close-packed structures. If the electrostatic repulsive interactions between particles were not strong enough to prevent close packing, the spherical particles would always tend to assemble into hexagonally close-packed arrays to make the system more thermodynamically stable [41]. After the solid water was removed by freeze-drying, a porous block comprising of loosely packed colloidal crystal grains was generated. The hexagonally ordered arrangement of the particles in each colloidal crystal grain was not disturbed because the ice sublimated directly from solid state to gaseous state in this process.

Based on the mechanism proposed above, the particles in the colloidal suspension successively experienced being concentrated, precipitating, forming colloidal crystal seeds, growing large, and finally forming colloidal crystal grains. Hexagonally close-packed arrangement was the thermodynamically favored array fashion obtained when the interparticle repulsive interactions were not too strong. Thus, factors including the initial volume fraction and the surface-charge magnitude of the particles should have no obvious effects on the array fashion of the particles and thus the peak positions of their reflectance spectra. Thus, the only necessary action is to make sure the particles are well dispersed in the precursor. To verify the inference, the influences of the volume fraction and the surface charge of the particles in precursor on the reflectance spectra of the colloidal crystal films were investigated.

**Figure 5:**
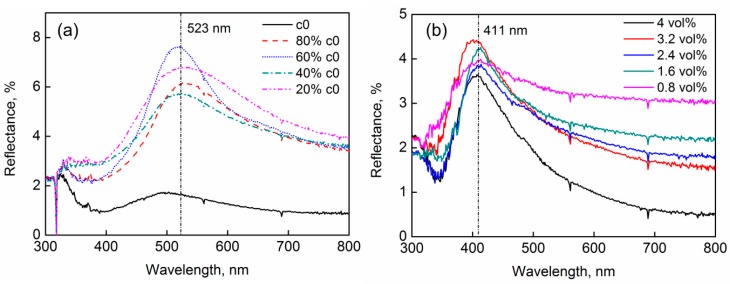
Reflection spectra of the colloidal crystal films prepared from: (**a**) SiO_2_ particle (diameter 240 nm)/water latex with different volume fractions of particles; and (**b**) PS particle (diameter 204 nm)/water latex with different volume fractions of particle. The incident angles were 15° in measurements.

SiO_2_ colloidal suspensions with five different volume fractions of particles were prepared and used to fabricate colloidal crystal films. The reflectance of the films was measured at a fixed incident angle of 15°. Based on the spectra shown in Figure 5a, it was found that the volume fraction of particles did not obviously influence the peak position. Only the peak values were influenced. All spectra had a peak value at the wavelength near 523 nm, which was quite close to the theoretical value 518.4 nm calculated with Equation (1) when assuming the particles were hexagonally closely arranged. Similar results were obtained when using polystyrene (PS) particles to fabricated the colloidal crystal films, as shown in Figure 5b. All spectra shown in this figure had peak values at the same wavelength near 411 nm, which was close to the theoretical value 427 nm calculated when assuming the particles are hexagonally closely packed. These results agree well with our inference presented above. The results further verify that the particles were hexagonally closely packed in the elemental grains that constituted the colloidal crystal film, and the reflectance peak of the films resulted from the light diffraction of the hexagonally close-packed array.

**Figure 6:**
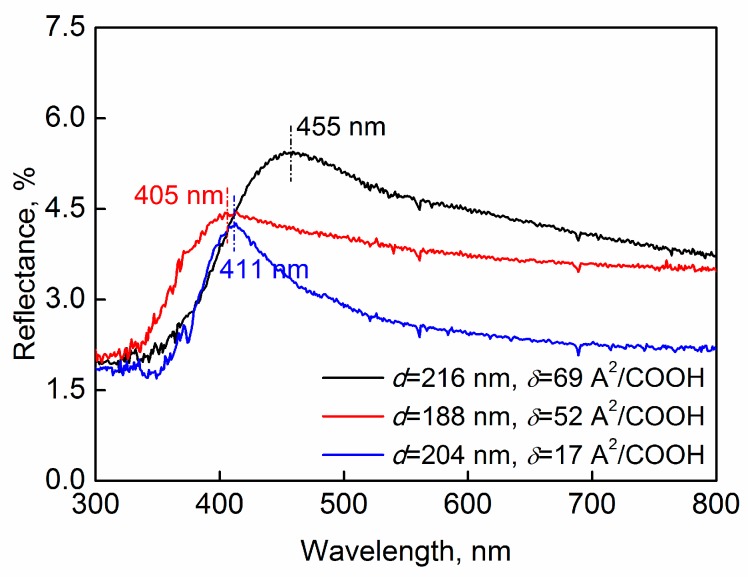
Reflectance spectra of the colloidal crystal films prepared from COOH–PS particle/water latex with different particle surface charges. The measurement incident angle was 15°.

The influence of the particle surface charge was also investigated, where the magnitude of the charge was tuned by adjusting the number of carboxyl groups covering the particle surface. Three batches of COOH–PS particles/water suspensions with different surface-charge magnitude were used to fabricate colloidal crystal films. The reflectance spectra of the films are shown in Figure 6. The three spectra had peak values at the wavelengths of 405, 411 and 455 nm, respectively. Since there were deviations in particle diameters among the three batches, which may lead to variations in the peak position, their influence should be considered. According to Equation (1), the theoretical peak values should be at the wavelengths of 394, 427 and 452 nm at the incident angle of 15° when the particle diameter is 188, 204, and 216 nm, respectively (if the particles are hexagonally closely packed). The measured values shown in Figure 6 were quite close to the theoretical ones, implying that the particles in every batch were hexagonally closely packed in their final produced films. In other words, the variations of surface charge in the scope of our current investigations did not obviously change the “hexagonally close-packed” arrangement of the particles, which means that the surface charge had no obvious effect on the array fashion. This is also coincident with our inference presented above. Note that this does not mean that the surface charge has no effect on the colloidal crystallization process, as it might influence the order degree of the elemental colloidal crystal grains.

In addition, factors such as initial particle concentration, surface charge of the particle and solidifying speed of the liquid water may influence the order degree or average size of the elemental colloidal crystal grains, which would consequently affect the peak value and bandgap width of the reflection spectrum of the colloidal crystal film. This will be investigated in detail in our further work. Since there are many tiny colloidal crystals with size of tens of micrometers or even a few micrometers, which would lead to strong light scattering, the produced powders (Figure 4d) had a pale appearance under white illumination, as shown in Figure 2a,b. The scattering may also be suppressed by optimizing the operating conditions to improve the order degree and geometrical sizes of the elemental colloidal crystal grains. As an alternative, a small amount of carbon can be added to absorb the incoherently scattered light [23,42], which may also enhance the structural color intensity.

## 4. Conclusions

Colloidal crystal powders and films with small free-standing colloidal crystal grains as building blocks were prepared based on the crystallization of monodispersed nanoparticles in particle/water suspension. The crystallization was induced by the freezing of liquid water, which made the solution be supersaturated with nanoparticles. The particles were hexagonally closely packed in their final arrangement and the structure was fixed by removing the solid water through freeze-drying. The initial volume fraction and surface-charge magnitude of the particles in the colloidal suspension showed no influence on the hexagonally close-packed array fashion of the particles. The strategy proposed here is an extremely convenient and highly efficient way to fabricate colloidal crystals, which has no need of careful control over the manipulation. In addition, it is intrinsically different from those conventional methods based on shearing force, surface tension, Columbic interaction or magnetic interaction. Since the crystallization of particles is induced by the solidification of water, various other kinds of solvents or monomers that can be solidified by lowering temperature or initiating polymerization may also be applied to fabricate other kinds of colloidal crystals. Thus, besides convenience and high-efficiency, the new strategy under study to prepare colloidal crystals is also versatile.

## References

[1] John S. (1987). Strong localization of photons in certain disordered dielectric superlattices. Phys. Rev. Lett..

[2] Yablonovitch E. (1987). Inhibited spontaneous emission in solid-state physics and electronics. Phys. Rev. Lett..

[3] Yablonovitch E., Gmitter T.J., Leung K.M. (1991). Photonic band structure: The face-centered-cubic case employing nonspherical atoms. Phys. Rev. Lett..

[4] Schroden R.C., Al-Daous M., Blanford C.F., Stein A. (2002). Optical properties of inverse opal photonic crystals. Chem. Mater..

[5] Meseguer F. (2005). Colloidal crystals as photonic crystals. Colloids Surf. A Physicochem. Eng. Asp..

[6] Zhang J., Sun Z., Yang B. (2009). Self-assembly of photonic crystals from polymer colloids. Curr. Opin. Colloid Interface Sci..

[7] Kim S.-H., Lee S.Y., Yang S.-M., Yi G.-R. (2011). Self-assembled colloidal structures for photonics. NPG Asia Mater..

[8] Yethiraj A., van Blaaderen A. (2003). A colloidal model system with an interaction tunable from hard sphere to soft and dipolar. Nature.

[9] Li F., Josephson D.P., Stein A. (2011). Colloidal assembly: The road from particles to colloidal molecules and crystals. Angew. Chem. Int. Ed..

[10] Sun C., Zhao X.-W., Zhao Y.-J., Zhu R., Gu Z.-Z. (2008). Fabrication of colloidal crystal beads by a drop-breaking technique and their application as bioassays. Small.

[11] Zhong K., Khorshid M., Li J., Markey K., Wagner P.H., Kai S., Vancleuvenbergen S., Clays K. (2016). Fabrication of optomicrofluidics for real-time bioassays based on hollow sphere colloidal photonic crystals with wettability patterns. J. Mater. Chem. C.

[12] Ding T., Smoukov S.K., Baumberg J.J. (2015). Stamping colloidal photonic crystals: A facile way towards complex pixel colour patterns for sensing and displays. Nanoscale.

[13] Zhong K., Wang L., Li J., Van C.S., Bartic C., Song K., Clays K. (2017). Real-time fluorescence detection in aqueous systems by combined and enhanced photonic and surface effects in patterned hollow sphere colloidal photonic crystals. Langmuir.

[14] Yang D., Qin Y., Ye S., Ge J. (2014). Polymerization-Induced Colloidal Assembly and Photonic Crystal Multilayer for Coding and Decoding. Adv. Funct. Mater..

[15] Zhong K., Li J., Liu L., Van Cleuvenbergen S., Song K., Clays K. (2018). Instantaneous, simple, and reversible revealing of Invisible patterns encrypted in robust hollow sphere colloidal photonic crystals. Adv. Mater..

[16] Parchine M., Kohoutek T., Bardosova M., Pemble M.E. (2018). Large area colloidal photonic crystals for light trapping in flexible organic photovoltaic modules applied using a roll-to-roll Langmuir-Blodgett method. Sol. Energy Mater. Sol. Cells.

[17] Kohoutek T., Parchine M., Bardosova M., Fudouzi H., Pemble M. (2018). Large-area flexible colloidal photonic crystal film stickers for light trapping applications. Opt. Mater. Express.

[18] Lee Y.-J., Braun P.V. (2003). Tunable inverse opal hydrogel pH sensors. Adv. Mater..

[19] Lee Y.-J., Pruzinsky S.A., Braun P.V. (2004). Glucose-sensitive inverse opal hydrogels: Analysis of optical diffraction response. Langmuir.

[20] Shin J., Braun P.V., Lee W. (2010). Fast response photonic crystal pH sensor based on templated photo-polymerized hydrogel inverse opal. Sens. Actuators B.

[21] Yan H., Blanford C.F., Smyrl W.H., Stein A. (2000). Preparation and structure of 3D ordered macroporous alloys by PMMA colloidal crystal templating. Chem. Commun..

[22] Lee W., Chan A., Bevan M.A., Lewis J.A., Braun P.V. (2004). Nanoparticle-mediated epitaxial assembly of colloidal crystals on patterned substrates. Langmuir.

[23] Josephson D.P., Popczun E.J., Stein A. (2013). Effects of integrated carbon as a light absorber on the coloration of photonic crystal-based pigments. J. Phys. Chem. C.

[24] Lai C., Wang Y., Wu C.-L., Zeng J., Lin C.-F. (2015). Preparation of a colloidal photonic crystal containing CuO nanoparticles with tunable structural colors. RSC Adv..

[25] Hu Y., Wang J., Li C., Wang Q., Wang H., Zhu J., Yang Y. (2013). Janus photonic crystal microspheres: Centrifugation-assisted generation and reversible optical property. Langmuir.

[26] Jiang P., Bertone J.F., Hwang K.S., Colvin V.L. (1999). Single-crystal colloidal multilayers of controlled thickness. Chem. Mater..

[27] Vlasov Y.A., Bo X.-Z., Sturm J.C., Norris D.J. (2001). On-chip natural assembly of silicon photonic bandgap crystals. Nature.

[28] Mishchenko L., Hatton B., Kolle M., Aizenberg J. (2012). Patterning hierarchy in direct and inverse opal crystals. Small.

[29] Schaffner M., England G., Kolle M., Aizenberg J., Vogel N. (2015). Combing bottom-up self-assembly with top-down microfabrication to create hierarchical inverse opals with high structural order. Small.

[30] Cong H., Cao W. (2003). Colloidal crystallization induced by capillary force. Langmuir.

[31] Hong S.-H., Moon J.H., Lim J.-M., Kim S.-H., Yang S.-M. (2005). Fabrication of spherical colloidal crystals using electrospray. Langmuir.

[32] Kim S.H., Hollingsworth A.D., Sacanna S., Chang S.J., Lee G., Pine D.J., Yi G.R. (2012). Synthesis and assembly of colloidal particles with sticky dimples. J. Am. Chem. Soc..

[33] Yin S.-N., Wang C.-F., Liu S.-S., Chen S. (2013). Facile fabrication of tunable colloidal photonic crystal hydrogel supraballs toward a colorimetric humidity sensor. J. Mater. Chem. C.

[34] Parchine M., Mcgrath J., Bardosova M., Pemble M.E. (2016). Large Area 2D and 3D Colloidal Photonic Crystals Fabricated by a Roll-to-Roll Langmuir-Blodgett Method. Langmuir.

[35] Askar K., Leo S.Y., Xu C., Liu D., Jiang P. (2016). Rapid electrostatics-assisted layer-by-layer assembly of near-infrared-active colloidal photonic crystals. J. Colloid Interf. Sci..

[36] Schäfer C.G., Lederle C., Zentel K., Stühn B., Gallei M. (2014). Utilizing stretch-tunable thermochromic elastomeric opal films as novel reversible switchable photonic materials. Macromol. Rapid Commun..

[37] Schäfer C.G., Winter T., Heidt S., Dietz C., Ding T., Baumberg J.J., Gallei M. (2015). Smart polymer inverse-opal photonic crystal films by melt-shear organization for hybrid core-shell architectures. J. Mater. Chem. C.

[38] Vowinkel S., Schäfer C.G., Cherkashinin G., Fasel C., Roth F., Liu N., Dietz C., Ionescu E., Gallei M. (2016). 3D-ordered carbon materials by melt-shear organization for tailor-made hybrid core–shell polymer particle architectures. J. Mater. Chem. C.

[39] Kim J., Song Y., He L., Kim H., Lee H., Park W., Yin Y., Kwon S. (2011). Real-time optofluidic synthesis of magnetochromatic microspheres for reversible structural color patterning. Small.

[40] You A., Cao Y., Cao G. (2015). Facile fabrication of magnetically assembled colloidal photonic crystal film via radical polymerization. RSC Adv..

[41] Velev O.D., Lenhoff A.M., Kaler E.W. (2000). A class of microstructured particles through colloidal crystallization. Science.

[42] Josephson D.P., Miller M., Stein A. (2014). Inverse opal SiO_2_ photonic crystals as structurally-colored pigments with additive primary colors. Z. Anorg. Allg. Chem..

